# Cyclo­octanaminium hydrogen succinate monohydrate

**DOI:** 10.1107/S1600536812011208

**Published:** 2012-03-28

**Authors:** Sanaz Khorasani, Manuel A. Fernandes

**Affiliations:** a Molecular Sciences Institute, School of Chemistry, University of the Witwatersrand, PO Wits 2050, Johannesburg, South Africa

## Abstract

In the title hydrated salt, C_8_H_18_N^+^·C_4_H_5_O_4_^−^·H_2_O, the cyclo­octyl­ ring of the cation is disordered over two positions in a 0.833 (3):0.167 (3) ratio. The structure contains various O—H.·O and N—H⋯O inter­actions, forming a hydrogen-bonded layer of mol­ecules perpendicular to the *c* axis. In each layer, the ammonium cation hydrogen bonds to two hydrogen succinate anions and one water mol­ecule. Each hydrogen succinate anion hydrogen bonds to neighbouring anions, forming a chain of mol­ecules along the *b* axis. In addition, each hydrogen succinate anion hydrogen bonds to two water mol­ecules and the ammonium cation.

## Related literature

For studies involving hydrogen-bonding inter­actions, see: Latimer & Rodebush (1920 ▶); Pimentel & McClellan (1960 ▶); Lemmerer (2011*a* ▶,*b* ▶). For graph-set motifs, see: Bernstein *et al.* (1995 ▶); Etter *et al.* (1990 ▶).
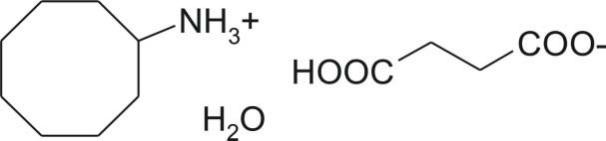


## Experimental

### 

#### Crystal data

C_8_H_18_N^+^·C_4_H_5_O_4_^−^·H_2_O
*M* *_r_* = 263.33
Orthorhombic, 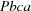

*a* = 8.4221 (6) Å
*b* = 14.3704 (9) Å
*c* = 23.7031 (16) Å
*V* = 2868.8 (3) Å^3^
*Z* = 8
Mo *K*α radiation
μ = 0.09 mm^−1^
*T* = 173 K
0.46 × 0.42 × 0.10 mm

#### Data collection

Bruker APEXII CCD diffractometer
12354 measured reflections
3461 independent reflections
2245 reflections with *I* > 2σ(*I*)
*R*_int_ = 0.038

#### Refinement

*R*[*F* ^2^ > 2σ(*F* ^2^)] = 0.050
*wR*(*F* ^2^) = 0.145
*S* = 1.04
3461 reflections
198 parameters
30 restraints
H atoms treated by a mixture of independent and constrained refinement
Δρ_max_ = 0.64 e Å^−3^
Δρ_min_ = −0.40 e Å^−3^

### 

Data collection: *APEX2* (Bruker, 2005 ▶); cell refinement: *SAINT-NT* (Bruker, 2005 ▶); data reduction: *SAINT-NT*; program(s) used to solve structure: *SHELXS97* (Sheldrick, 2008 ▶); program(s) used to refine structure: *SHELXL97* (Sheldrick, 2008 ▶); molecular graphics: *ORTEP-3 for Windows* (Farrugia, 1997 ▶) and *SCHAKAL99* (Keller, 1999 ▶); software used to prepare material for publication: *WinGX* (Farrugia, 1999 ▶) and *PLATON* (Spek, 2009 ▶).

## Supplementary Material

Crystal structure: contains datablock(s) global, I. DOI: 10.1107/S1600536812011208/sj5211sup1.cif

Structure factors: contains datablock(s) I. DOI: 10.1107/S1600536812011208/sj5211Isup2.hkl

Supplementary material file. DOI: 10.1107/S1600536812011208/sj5211Isup3.cml

Additional supplementary materials:  crystallographic information; 3D view; checkCIF report

## Figures and Tables

**Table 1:** Hydrogen-bond geometry (Å, °)

*D*—H⋯*A*	*D*—H	H⋯*A*	*D*⋯*A*	*D*—H⋯*A*
O3—H3⋯O1^i^	0.84	1.72	2.5586 (18)	179
N1—H1*A*⋯O2^ii^	0.91	1.93	2.834 (2)	175
N1—H1*B*⋯O1*W*^iii^	0.91	1.91	2.804 (2)	168
N1—H1*C*⋯O1	0.91	1.89	2.7866 (19)	168
N1—H1*A*⋯O2^ii^	0.91	1.93	2.834 (2)	175
O1*W*—H1*WA*⋯O2	0.83 (3)	1.99 (3)	2.807 (2)	167 (3)
O1*W*—H1*WB*⋯O4^iv^	0.85 (3)	2.03 (3)	2.855 (2)	165 (2)

Symmetry codes: (i) 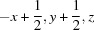; (ii) 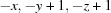; (iii) 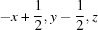; (iv) 
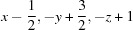
.

**Fig. 1.**
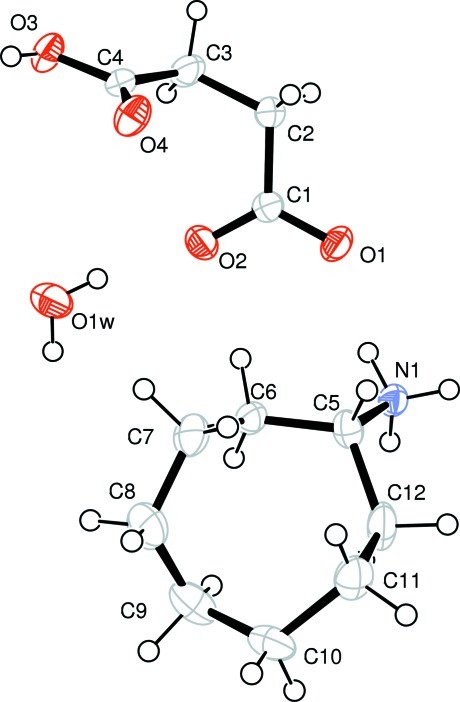
The asymmetric unit of (I). Only the major disorder component of the cation is shown.

**Fig. 2.**
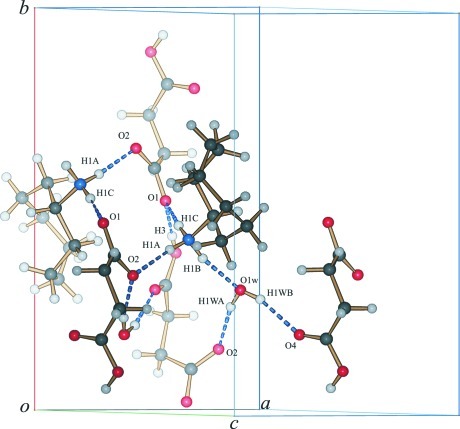
O—H···O, N—H···O hydrogen bonding interactions in the structure of (I) drawn as dashed lines.

**Fig. 3.**
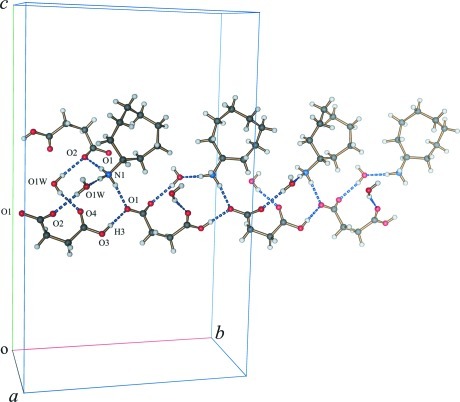
Hydrogen bonded chains of hydrogen succinate anions in the structure of (I). Also shown are the hydrogen bonding environments around the ammonium cations and the water molecules. Hydrogen bonds are drawn as dashed lines.

## supplementary crystallographic information

### Comment

Intramolecular and intermolecular hydrogen bonding is of great importance in chemical and biological systems. As a consequence it has been studied extensively since the 1920's (Latimer & Rodebush, 1920; Pimentel & McClellan, 1960) and is still an area of intense interest. In the crystal engineering field, hydrogen bonding plays an important role in organizing molecules, assembling them to create supramolecules and controlling their dimensions in one-, two- or three-dimensions. This is a requirement in order to create functional materials by design. Ammonium carboxylate salts, by having strong charge-assisted N—H..O hydrogen bonds, can be used to align molecules in desired directions, which is also useful for creating functional materials (Lemmerer, 2011*a*; Lemmerer, 2011*b*).

The title compound (Fig.1) crystallizes in *Pbca* and contains three independent molecules: a cyclooctanaminium cation disordered over two positions in a 0833 (3):0.167 (3) ratio, a hydrogen succinate anion, and a water molecule (Scheme 1). The crystal structure consists of a hydrogen bonded layer composed of several different hydrogen bonds between the three molecules (Fig. 2). The hydrogen succinate anions are linked *via* an intermolecular O3—H3···O1 hydrogen bond to form chains of molecules along the *b* axis described by the graph set C7 (Fig. 3) (Etter *et al.*, 1990; Bernstein *et al.*, 1995). All three independent molecules are linked *via* hydrogen bonding to form a ring described by the graph set motif *R*^3^_5_(12). The three ammonium hydrogen atoms are involved in strong hydrogen bonds with the O atoms of the neighbouring succinate anions (N—H1C···O1 and N—H1A···O2) and a hydrogen bond with the water molecule (N—H1B···O1W). The water molecule act as both hydrogen acceptor (accepts the H atom from N) and donor (donates H atoms to the succinate anions) to surrounding molecules. The combination of these hydrogen bonds leads to a two-dimensional hydrogen bonded layer of molecules perpedicular to the *c* axis. A list hydrogen bonding interactions are given in Table 1.

### Experimental

The title compound was obtained after a failed synthesis. Succinic acid [succinic anhydride having reacted with water in the reagent bottle over time (years)] was dissolved in dioxane followed by the addition of an equimolar amount of cyclooctylamine. After 6 h, thionyl chloride in dioxane was slowly added to the reaction mixture at room temperature. The mixture was then kept at 50 °C for 6 h, followed by neutralization of excess thionyl chloride by pouring the mixture into a beaker containing ice. The mixture was then filtered and the solvent removed under reduced pressure. This was then redissolved in methanol which after a few days of evaporation yielded crystals suitable for analysis by X-ray diffraction.

### Refinement

H atoms in the cation and anion were positioned geometrically, and allowed to ride on their parent atoms, with Atom—H bond lengths of 0.99 Å (CH_2_), or 0.91 Å (NH_3_), or 0.84 Å (COOH), and isotropic displacement parameters set to 1.2 times (CH_2_) or 1.5 times (NH_3_ and COOH) the *U*_eq_ of the parent atom. Hydrogen atoms of the water molecule were refined freely.

### Crystal data

**Table:**

C_8_H_18_N^+^·C_4_H_5_O_4_^−^·H_2_O	*F*(000) = 1152
*M**_r_* = 263.33	*D*_x_ = 1.219 Mg m^−^^3^
Orthorhombic, *P**b**c**a*	Mo *K*α radiation, λ = 0.71073 Å
Hall symbol: -P 2ac 2ab	Cell parameters from 2803 reflections
*a* = 8.4221 (6) Å	θ = 2.9–27.8°
*b* = 14.3704 (9) Å	µ = 0.09 mm^−^^1^
*c* = 23.7031 (16) Å	*T* = 173 K
*V* = 2868.8 (3) Å^3^	Block, colourless
*Z* = 8	0.46 × 0.42 × 0.10 mm

### Data collection

**Table:**

Bruker APEXII CCD diffractometer	2245 reflections with *I* > 2σ(*I*)
Radiation source: fine-focus sealed tube	*R*_int_ = 0.038
Graphite monochromator	θ_max_ = 28.0°, θ_min_ = 1.7°
φ and ω scans	*h* = −9→11
12354 measured reflections	*k* = −18→18
3461 independent reflections	*l* = −15→31

### Refinement

**Table:**

Refinement on *F*^2^	Primary atom site location: structure-invariant direct methods
Least-squares matrix: full	Secondary atom site location: difference Fourier map
*R*[*F*^2^ > 2σ(*F*^2^)] = 0.050	Hydrogen site location: inferred from neighbouring sites
*wR*(*F*^2^) = 0.145	H atoms treated by a mixture of independent and constrained refinement
*S* = 1.04	*w* = 1/[σ^2^(*F*_o_^2^) + (0.0721*P*)^2^ + 0.4241*P*] where *P* = (*F*_o_^2^ + 2*F*_c_^2^)/3
3461 reflections	(Δ/σ)_max_ < 0.001
198 parameters	Δρ_max_ = 0.64 e Å^−^^3^
30 restraints	Δρ_min_ = −0.40 e Å^−^^3^

### Special details

**Table:**

Geometry. All e.s.d.'s (except the e.s.d. in the dihedral angle between two l.s. planes) are estimated using the full covariance matrix. The cell e.s.d.'s are taken into account individually in the estimation of e.s.d.'s in distances, angles and torsion angles; correlations between e.s.d.'s in cell parameters are only used when they are defined by crystal symmetry. An approximate (isotropic) treatment of cell e.s.d.'s is used for estimating e.s.d.'s involving l.s. planes.
Refinement. Refinement of *F*^2^ against ALL reflections. The weighted *R*-factor *wR* and goodness of fit *S* are based on *F*^2^, conventional *R*-factors *R* are based on *F*, with *F* set to zero for negative *F*^2^. The threshold expression of *F*^2^ > σ(*F*^2^) is used only for calculating *R*-factors(gt) *etc*. and is not relevant to the choice of reflections for refinement. *R*-factors based on *F*^2^ are statistically about twice as large as those based on *F*, and *R*- factors based on ALL data will be even larger.

### Fractional atomic coordinates and isotropic or equivalent isotropic displacement parameters (Å^2^)

**Table:**

	*x*	*y*	*z*	*U*_iso_*/*U*_eq_	Occ. (<1)
C1	0.1688 (2)	0.60717 (12)	0.41032 (7)	0.0245 (4)	
C2	0.2573 (3)	0.64226 (13)	0.35928 (8)	0.0369 (5)	
H2A	0.2416	0.5976	0.3280	0.044*	
H2B	0.3722	0.6435	0.3682	0.044*	
C3	0.2077 (3)	0.73821 (13)	0.33930 (8)	0.0379 (5)	
H3A	0.2565	0.7501	0.3020	0.046*	
H3B	0.0910	0.7391	0.3343	0.046*	
C4	0.2540 (2)	0.81521 (12)	0.37880 (7)	0.0259 (4)	
O1	0.20323 (19)	0.52624 (8)	0.42678 (5)	0.0377 (4)	
O2	0.06634 (15)	0.65701 (9)	0.43292 (5)	0.0321 (3)	
O3	0.19460 (16)	0.89565 (8)	0.36302 (5)	0.0331 (3)	
H3	0.2287	0.9378	0.3843	0.050*	
O4	0.33717 (17)	0.80532 (9)	0.41988 (5)	0.0351 (3)	
C5	0.2848 (3)	0.49102 (16)	0.57502 (9)	0.0277 (5)	0.833 (3)
H5	0.3998	0.5017	0.5667	0.033*	0.833 (3)
C6	0.1996 (3)	0.58377 (14)	0.57461 (9)	0.0288 (5)	0.833 (3)
H6A	0.0873	0.5735	0.5852	0.035*	0.833 (3)
H6B	0.2009	0.6087	0.5357	0.035*	0.833 (3)
C7	0.2706 (8)	0.6568 (3)	0.61424 (15)	0.0391 (10)	0.833 (3)
H7A	0.3827	0.6399	0.6215	0.047*	0.833 (3)
H7B	0.2706	0.7174	0.5944	0.047*	0.833 (3)
C8	0.1887 (4)	0.66935 (19)	0.67014 (11)	0.0491 (7)	0.833 (3)
H8A	0.2681	0.6949	0.6968	0.059*	0.833 (3)
H8B	0.1065	0.7179	0.6650	0.059*	0.833 (3)
C9	0.1127 (4)	0.5907 (2)	0.69798 (13)	0.0453 (8)	0.833 (3)
H9A	0.0406	0.5614	0.6702	0.054*	0.833 (3)
H9B	0.0450	0.6160	0.7285	0.054*	0.833 (3)
C10	0.2099 (6)	0.5162 (3)	0.72271 (15)	0.0524 (11)	0.833 (3)
H10A	0.1372	0.4676	0.7372	0.063*	0.833 (3)
H10B	0.2668	0.5425	0.7556	0.063*	0.833 (3)
C11	0.3367 (3)	0.46707 (17)	0.68355 (10)	0.0395 (7)	0.833 (3)
H11A	0.4219	0.5121	0.6746	0.047*	0.833 (3)
H11B	0.3853	0.4148	0.7045	0.047*	0.833 (3)
C12	0.2701 (6)	0.4314 (2)	0.63067 (12)	0.0482 (10)	0.833 (3)
H12A	0.1557	0.4197	0.6372	0.058*	0.833 (3)
H12B	0.3202	0.3703	0.6232	0.058*	0.833 (3)
C5B	0.2128 (17)	0.4898 (8)	0.5839 (5)	0.0297 (15)*	0.167 (3)
H5B	0.1059	0.5155	0.5936	0.036*	0.167 (3)
C6B	0.3370 (13)	0.5619 (7)	0.5758 (4)	0.0297 (15)*	0.167 (3)
H6C	0.3510	0.5740	0.5350	0.036*	0.167 (3)
H6D	0.4392	0.5388	0.5910	0.036*	0.167 (3)
C7B	0.291 (4)	0.6541 (14)	0.6063 (7)	0.0297 (15)*	0.167 (3)
H7C	0.3672	0.7026	0.5938	0.036*	0.167 (3)
H7D	0.1852	0.6729	0.5924	0.036*	0.167 (3)
C8B	0.2862 (16)	0.6565 (8)	0.6697 (4)	0.0297 (15)*	0.167 (3)
H8C	0.2668	0.7219	0.6809	0.036*	0.167 (3)
H8D	0.3938	0.6402	0.6833	0.036*	0.167 (3)
C9B	0.1692 (17)	0.5967 (9)	0.7026 (6)	0.0297 (15)*	0.167 (3)
H9C	0.1373	0.6298	0.7375	0.036*	0.167 (3)
H9D	0.0728	0.5865	0.6796	0.036*	0.167 (3)
C10B	0.241 (3)	0.5046 (11)	0.7179 (8)	0.0297 (15)*	0.167 (3)
H10C	0.1953	0.4833	0.7541	0.036*	0.167 (3)
H10D	0.3567	0.5124	0.7232	0.036*	0.167 (3)
C11B	0.2108 (14)	0.4281 (7)	0.6716 (4)	0.0297 (15)*	0.167 (3)
H11C	0.2255	0.3688	0.6922	0.036*	0.167 (3)
H11D	0.0953	0.4325	0.6642	0.036*	0.167 (3)
C12B	0.269 (3)	0.4122 (11)	0.6238 (6)	0.0297 (15)*	0.167 (3)
H12C	0.2338	0.3507	0.6098	0.036*	0.167 (3)
H12D	0.3869	0.4118	0.6260	0.036*	0.167 (3)
N1	0.21505 (19)	0.43116 (10)	0.52915 (6)	0.0265 (3)	
H1A	0.1213	0.4064	0.5409	0.040*	0.833 (3)
H1B	0.2846	0.3845	0.5212	0.040*	0.833 (3)
H1C	0.1984	0.4659	0.4976	0.040*	0.833 (3)
H1D	0.2341	0.4641	0.4971	0.040*	0.167 (3)
H1E	0.1091	0.4198	0.5322	0.040*	0.167 (3)
H1F	0.2687	0.3762	0.5278	0.040*	0.167 (3)
O1W	0.0370 (2)	0.80506 (10)	0.50947 (7)	0.0424 (4)	
H1WA	0.061 (4)	0.764 (2)	0.4866 (12)	0.071 (9)*	
H1WB	−0.029 (3)	0.7817 (17)	0.5325 (11)	0.060 (8)*	

### Atomic displacement parameters (Å^2^)

**Table:**

	*U*^11^	*U*^22^	*U*^33^	*U*^12^	*U*^13^	*U*^23^
C1	0.0283 (10)	0.0228 (8)	0.0223 (8)	−0.0050 (8)	−0.0029 (7)	−0.0023 (6)
C2	0.0552 (13)	0.0230 (9)	0.0327 (10)	0.0022 (9)	0.0148 (9)	−0.0003 (7)
C3	0.0630 (15)	0.0269 (9)	0.0239 (9)	−0.0035 (10)	0.0035 (9)	0.0029 (7)
C4	0.0287 (9)	0.0231 (8)	0.0258 (8)	−0.0011 (7)	0.0050 (8)	0.0049 (7)
O1	0.0629 (10)	0.0207 (6)	0.0294 (7)	0.0024 (6)	0.0016 (7)	0.0029 (5)
O2	0.0326 (7)	0.0308 (7)	0.0331 (7)	−0.0005 (6)	0.0065 (6)	−0.0058 (5)
O3	0.0427 (8)	0.0220 (6)	0.0345 (7)	0.0010 (6)	−0.0086 (6)	0.0048 (5)
O4	0.0384 (8)	0.0296 (7)	0.0373 (7)	0.0021 (6)	−0.0091 (6)	0.0068 (5)
C5	0.0279 (12)	0.0276 (11)	0.0277 (11)	0.0047 (11)	−0.0060 (10)	−0.0061 (8)
C6	0.0406 (14)	0.0232 (10)	0.0225 (10)	0.0022 (10)	−0.0017 (9)	0.0011 (8)
C7	0.054 (3)	0.0220 (11)	0.0417 (19)	−0.0071 (13)	0.0006 (16)	−0.0053 (12)
C8	0.070 (2)	0.0410 (14)	0.0369 (14)	0.0016 (14)	−0.0018 (14)	−0.0144 (11)
C9	0.0325 (16)	0.0675 (18)	0.0361 (14)	0.0066 (14)	0.0040 (13)	−0.0117 (12)
C10	0.067 (3)	0.063 (2)	0.0265 (14)	0.0106 (18)	0.0140 (15)	0.0001 (13)
C11	0.0496 (16)	0.0371 (13)	0.0319 (12)	0.0087 (12)	−0.0139 (11)	0.0000 (10)
C12	0.081 (2)	0.0314 (17)	0.0318 (15)	0.0261 (18)	−0.0134 (15)	−0.0079 (11)
N1	0.0344 (9)	0.0213 (7)	0.0236 (7)	0.0021 (7)	−0.0010 (6)	−0.0009 (5)
O1W	0.0498 (10)	0.0402 (8)	0.0372 (8)	−0.0188 (8)	0.0151 (7)	−0.0122 (7)

### Geometric parameters (Å, º)

**Table:**

C1—O2	1.243 (2)	C12—H12A	0.9900
C1—O1	1.260 (2)	C12—H12B	0.9900
C1—C2	1.508 (3)	C5B—C6B	1.485 (13)
C2—C3	1.517 (3)	C5B—C12B	1.539 (15)
C2—H2A	0.9900	C5B—N1	1.547 (13)
C2—H2B	0.9900	C5B—H5B	1.0000
C3—C4	1.501 (3)	C6B—C7B	1.557 (17)
C3—H3A	0.9900	C6B—H6C	0.9900
C3—H3B	0.9900	C6B—H6D	0.9900
C4—O4	1.208 (2)	C7B—C8B	1.506 (16)
C4—O3	1.314 (2)	C7B—H7C	0.9900
O3—H3	0.8400	C7B—H7D	0.9900
C5—N1	1.506 (3)	C8B—C9B	1.522 (14)
C5—C6	1.514 (3)	C8B—H8C	0.9900
C5—C12	1.577 (4)	C8B—H8D	0.9900
C5—H5	1.0000	C9B—C10B	1.499 (16)
C6—C7	1.530 (5)	C9B—H9C	0.9900
C6—H6A	0.9900	C9B—H9D	0.9900
C6—H6B	0.9900	C10B—C11B	1.573 (16)
C7—C8	1.505 (4)	C10B—H10C	0.9900
C7—H7A	0.9900	C10B—H10D	0.9900
C7—H7B	0.9900	C11B—C12B	1.257 (15)
C8—C9	1.457 (4)	C11B—H11C	0.9900
C8—H8A	0.9900	C11B—H11D	0.9900
C8—H8B	0.9900	C12B—H12C	0.9900
C9—C10	1.470 (4)	C12B—H12D	0.9900
C9—H9A	0.9900	N1—H1A	0.9100
C9—H9B	0.9900	N1—H1B	0.9100
C10—C11	1.581 (4)	N1—H1C	0.9100
C10—H10A	0.9900	N1—H1D	0.9100
C10—H10B	0.9900	N1—H1E	0.9100
C11—C12	1.466 (4)	N1—H1F	0.9100
C11—H11A	0.9900	O1W—H1WA	0.83 (3)
C11—H11B	0.9900	O1W—H1WB	0.85 (3)
			
O2—C1—O1	123.91 (17)	C6B—C5B—H5B	113.9
O2—C1—C2	119.76 (16)	C12B—C5B—H5B	113.9
O1—C1—C2	116.32 (16)	N1—C5B—H5B	113.9
C1—C2—C3	114.74 (17)	C5B—C6B—C7B	111.1 (14)
C1—C2—H2A	108.6	C5B—C6B—H6C	109.4
C3—C2—H2A	108.6	C7B—C6B—H6C	109.4
C1—C2—H2B	108.6	C5B—C6B—H6D	109.4
C3—C2—H2B	108.6	C7B—C6B—H6D	109.4
H2A—C2—H2B	107.6	H6C—C6B—H6D	108.0
C4—C3—C2	113.84 (17)	C8B—C7B—C6B	119.3 (16)
C4—C3—H3A	108.8	C8B—C7B—H7C	107.5
C2—C3—H3A	108.8	C6B—C7B—H7C	107.5
C4—C3—H3B	108.8	C8B—C7B—H7D	107.5
C2—C3—H3B	108.8	C6B—C7B—H7D	107.5
H3A—C3—H3B	107.7	H7C—C7B—H7D	107.0
O4—C4—O3	123.63 (16)	C7B—C8B—C9B	121.2 (14)
O4—C4—C3	124.51 (16)	C7B—C8B—H8C	107.0
O3—C4—C3	111.86 (16)	C9B—C8B—H8C	107.0
C4—O3—H3	109.5	C7B—C8B—H8D	107.0
N1—C5—C6	108.26 (17)	C9B—C8B—H8D	107.0
N1—C5—C12	105.3 (2)	H8C—C8B—H8D	106.8
C6—C5—C12	116.5 (2)	C10B—C9B—C8B	111.2 (13)
N1—C5—H5	108.9	C10B—C9B—H9C	109.4
C6—C5—H5	108.9	C8B—C9B—H9C	109.4
C12—C5—H5	108.9	C10B—C9B—H9D	109.4
C5—C6—C7	114.5 (3)	C8B—C9B—H9D	109.4
C5—C6—H6A	108.6	H9C—C9B—H9D	108.0
C7—C6—H6A	108.6	C9B—C10B—C11B	112.6 (14)
C5—C6—H6B	108.6	C9B—C10B—H10C	109.1
C7—C6—H6B	108.6	C11B—C10B—H10C	109.1
H6A—C6—H6B	107.6	C9B—C10B—H10D	109.1
C8—C7—C6	116.3 (4)	C11B—C10B—H10D	109.1
C8—C7—H7A	108.2	H10C—C10B—H10D	107.8
C6—C7—H7A	108.2	C12B—C11B—C10B	133.8 (15)
C8—C7—H7B	108.2	C12B—C11B—H11C	103.8
C6—C7—H7B	108.2	C10B—C11B—H11C	103.8
H7A—C7—H7B	107.4	C12B—C11B—H11D	103.8
C9—C8—C7	120.5 (3)	C10B—C11B—H11D	103.8
C9—C8—H8A	107.2	H11C—C11B—H11D	105.4
C7—C8—H8A	107.2	C11B—C12B—C5B	107.4 (13)
C9—C8—H8B	107.2	C11B—C12B—H12C	110.2
C7—C8—H8B	107.2	C5B—C12B—H12C	110.2
H8A—C8—H8B	106.8	C11B—C12B—H12D	110.2
C8—C9—C10	120.0 (3)	C5B—C12B—H12D	110.2
C8—C9—H9A	107.3	H12C—C12B—H12D	108.5
C10—C9—H9A	107.3	C5—N1—H1A	109.9
C8—C9—H9B	107.3	C5B—N1—H1A	86.8
C10—C9—H9B	107.3	C5—N1—H1B	108.6
H9A—C9—H9B	106.9	C5B—N1—H1B	125.6
C9—C10—C11	117.9 (3)	H1A—N1—H1B	109.5
C9—C10—H10A	107.8	C5—N1—H1C	109.9
C11—C10—H10A	107.8	C5B—N1—H1C	112.9
C9—C10—H10B	107.8	H1A—N1—H1C	109.5
C11—C10—H10B	107.8	H1B—N1—H1C	109.5
H10A—C10—H10B	107.2	C5—N1—H1D	103.8
C12—C11—C10	113.6 (3)	C5B—N1—H1D	114.9
C12—C11—H11A	108.9	H1A—N1—H1D	127.8
C10—C11—H11A	108.9	H1B—N1—H1D	95.6
C12—C11—H11B	108.9	C5—N1—H1E	115.3
C10—C11—H11B	108.9	C5B—N1—H1E	91.1
H11A—C11—H11B	107.7	H1B—N1—H1E	121.0
C11—C12—C5	119.7 (3)	H1C—N1—H1E	90.8
C11—C12—H12A	107.4	H1D—N1—H1E	109.5
C5—C12—H12A	107.4	C5—N1—H1F	109.1
C11—C12—H12B	107.4	C5B—N1—H1F	120.6
C5—C12—H12B	107.4	H1A—N1—H1F	95.9
H12A—C12—H12B	106.9	H1C—N1—H1F	121.5
C6B—C5B—C12B	111.5 (12)	H1D—N1—H1F	109.5
C6B—C5B—N1	105.3 (9)	H1E—N1—H1F	109.5
C12B—C5B—N1	96.7 (9)	H1WA—O1W—H1WB	107 (2)
			
O2—C1—C2—C3	−1.1 (3)	C12B—C5B—C6B—C7B	−108.6 (15)
O1—C1—C2—C3	177.55 (17)	N1—C5B—C6B—C7B	147.6 (12)
C1—C2—C3—C4	69.2 (2)	C5B—C6B—C7B—C8B	68 (3)
C2—C3—C4—O4	7.5 (3)	C6B—C7B—C8B—C9B	−63 (3)
C2—C3—C4—O3	−173.33 (17)	C7B—C8B—C9B—C10B	91.6 (19)
N1—C5—C6—C7	−173.0 (2)	C8B—C9B—C10B—C11B	−90.2 (17)
C12—C5—C6—C7	68.7 (4)	C9B—C10B—C11B—C12B	76 (2)
C5—C6—C7—C8	−99.4 (4)	C10B—C11B—C12B—C5B	−70 (2)
C6—C7—C8—C9	32.6 (6)	C6B—C5B—C12B—C11B	100.1 (17)
C7—C8—C9—C10	70.8 (5)	N1—C5B—C12B—C11B	−150.5 (14)
C8—C9—C10—C11	−54.0 (5)	C6—C5—N1—C5B	−60.1 (11)
C9—C10—C11—C12	−53.1 (5)	C12—C5—N1—C5B	65.1 (12)
C10—C11—C12—C5	97.5 (4)	C6B—C5B—N1—C5	30.7 (7)
N1—C5—C12—C11	176.1 (3)	C12B—C5B—N1—C5	−83.8 (15)
C6—C5—C12—C11	−63.9 (5)		

### Hydrogen-bond geometry (Å, º)

**Table:**

*D*—H···*A*	*D*—H	H···*A*	*D*···*A*	*D*—H···*A*
O3—H3···O1^i^	0.84	1.72	2.5586 (18)	179
N1—H1*A*···O2^ii^	0.91	1.93	2.834 (2)	175
N1—H1*B*···O1*W*^iii^	0.91	1.91	2.804 (2)	168
N1—H1*C*···O1	0.91	1.89	2.7866 (19)	168
N1—H1*A*···O2^ii^	0.91	1.93	2.834 (2)	175
O1*W*—H1*WA*···O2	0.83 (3)	1.99 (3)	2.807 (2)	167 (3)
O1*W*—H1*WB*···O4^iv^	0.85 (3)	2.03 (3)	2.855 (2)	165 (2)

Symmetry codes: (i) −*x*+1/2, *y*+1/2, *z*; (ii) −*x*, −*y*+1, −*z*+1; (iii) −*x*+1/2, *y*−1/2, *z*; (iv) *x*−1/2, −*y*+3/2, −*z*+1.
